# Comparative Study of Metagenomics and Metatranscriptomics to Reveal Microbiomes in Overwintering Pepper Fruits

**DOI:** 10.3390/ijms22126202

**Published:** 2021-06-08

**Authors:** Yeonhwa Jo, Chang-Gi Back, Kook-Hyung Kim, Hyosub Chu, Jeong Hun Lee, Sang Hyun Moh, Won Kyong Cho

**Affiliations:** 1 Research Institute of Agriculture and Life Sciences, College of Agriculture and Life Sciences, Seoul National University, Seoul 08826, Korea; yeonhwajo@gmail.com (Y.J.); kookkim@snu.ac.kr (K.-H.K.); 2 Horticultural and Herbal Crop Environment Division, National Institute of Horticultural and Herbal Science, RDA, Wanju 55365, Korea; plantdoctor7@korea.kr; 3 R&D Division, BERTIS Inc., Seongnam-si 13605, Korea; hyosubchu@gmail.com; 4 Anti-Aging Research Institute of BIO-FD&C Co., Ltd., Incheon 21990, Korea; jhlee@biofdnc.com (J.H.L.); biofdnc@gmail.com (S.H.M.)

**Keywords:** pepper, fruit, microbiome, bacteria, fungi, viruses, metagenomics, metatranscriptomics

## Abstract

Red pepper (*Capsicum annuum*, L.), is one of the most important spice plants in Korea. Overwintering pepper fruits are a reservoir of various microbial pepper diseases. Here, we conducted metagenomics (DNA sequencing) and metatranscriptomics (RNA sequencing) using samples collected from three different fields. We compared two different library types and three different analytical methods for the identification of microbiomes in overwintering pepper fruits. Our results demonstrated that DNA sequencing might be useful for the identification of bacteria and DNA viruses such as bacteriophages, while mRNA sequencing might be beneficial for the identification of fungi and RNA viruses. Among three analytical methods, KRAKEN2 with raw data reads (KRAKEN2_R) might be superior for the identification of microbial species to other analytical methods. However, some microbial species with a low number of reads were wrongly assigned at the species level by KRAKEN2_R. Moreover, we found that the databases for bacteria and viruses were better established as compared to the fungal database with limited genome data. In summary, we carefully suggest that different library types and analytical methods with proper databases should be applied for the purpose of microbiome study.

## 1. Introduction

Red pepper (*Capsicum annuum*, L.), is one of the important spice plants belonging to the family *Solanaceae* found in many countries around the world such as China, Mexico, Turkey, Indonesia, India, Spain, United States, and Korea [1]. Infections of diverse plant pathogenic microorganisms such as bacteria, fungi, and viruses as well as insects in pepper results in a severe reduction of pepper quantity and quality [2]. Thus, a wide range of agricultural chemicals are routinely sprayed on growing pepper plants to yield high-quality pepper fruits in Korea. Farmers normally remove pepper plants at the end of fall after harvesting the red pepper fruits; however, some pepper plants overwinter. The overwintering pepper fruits in the field represent a reservoir of various diseases, not only for pepper plants, but also other economically important plants. Many fungi generate overwintering spores that can be viable for several years. Although practicing crop rotation is suggested for farmers, they like to grow pepper plants in the same spots for many years. This increases the probability of developing pathogenic microorganism and pest infestations.

Bacterial leaf spot disease caused by *Xanthomonas* species is very common, causing yellowish spots on pepper leaves [3,4]. The humid and warm climate during the summer season is optimal for the spread of pathogenic fungi. Anthraconse caused by *Colletotrichum* species, powdery mildew caused by *Leveillula taurica*, southern blight caused by *Sclerotinia* species, phytophthora blight caused by *Phytophthora* species, and gray mold caused by *Botrytis* species are the most well-known pathogenic fungi in red pepper growing [5,6,7,8,9,10,11,12,13]. Cucumber mosaic virus (CMV), pepper mottle virus (PepMoV), broad bean wilt virus 2 (BBWV2), and tomato spotted wilt virus (TSWV) are major viruses infecting pepper, causing chlorosis, light brown flecks, mosaic disease, and multiple ring spots on the infected leaves and fruits [14,15,16,17,18,19,20,21].

Traditionally, cultivation-dependent methods have been widely used to identify plant microbial pathogens [22]. Over the last twenty years, with the rapid development of next-generation sequencing (NGS), cultivation-independent methods such as metagenomics (DNA) and metatranscriptomics (RNA) have provided a deeper insight into the microbial pathogens in numerous crop plants. In metagenomics, the bacterial 16S ribosomal RNA (rRNA) gene and fungal internal transcribed spacer (ITS) region of rRNA are PCR-amplified from the extracted DNA and sequenced by NGS followed by bioinformatic analyses to identify microbial pathogens [23,24]. By contrast, DNA and RNA shotgun sequencing can reveal not only microbial communities but also all living organisms within the samples [25,26]. Viruses do not possess any conserved sequences like bacteria and fungi, therefore, RNA sequencing is frequently used to identify viruses infecting plants [27,28]. Although NGS followed by bioinformatic analyses can reveal the microbial community composition in detail, it might be difficult to distinguish viable microorganisms from NGS data. Recently, several techniques including microscopy, flow cytometry, and quantitative PCR (qPCR) have been developed to distinguish between live and dead microorganisms in natural environments [29].

Not only experimental approaches but also data analyses play an important role in the comprehensive and accurate identification of microbial pathogens in given samples. In this study, we revealed microbiomes of pepper fruits collected from three different fields by metagenomics and metatranscriptomics. In addition, we compared three different methods (KRAKEN2 [30] using raw data, MEGAN6 [31] using raw data, and MEGAN6 using assembled sequences) for the identification of microorganisms such as bacteria, fungi, and viruses.

## 2. Results

### 2.1. Metagenomics and Metatranscriptomics for Overwintering Pepper Fruits

**Figure 1:**
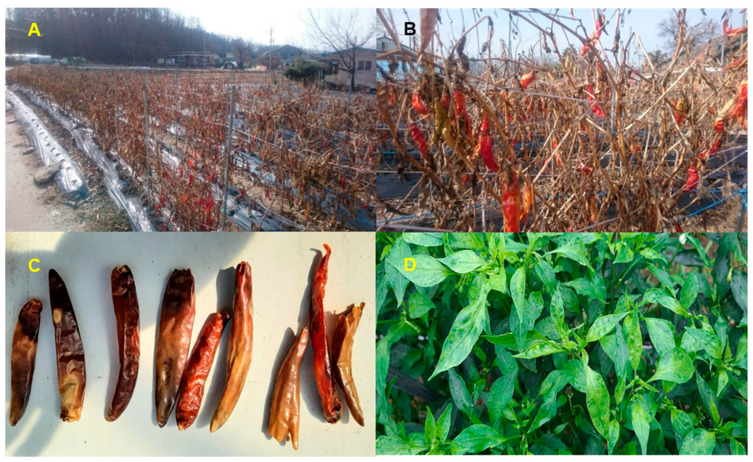
Images of pepper fruits in field. Overwintering pepper plants in Region 63 (**A**) and Region 89 (**B**). (**C**) Dried pepper fruits derived from greenhouse in Region 48. (**D**) Pepper plants showing viral disease symptoms.

In general, most pepper plants are removed from the field before the winter season, however, some pepper plants may remain in the field (Figure 1A,B). In this study, we harvested three different pepper fruits from three different geographical regions. Most overwintering pepper fruits displayed severe disease symptoms (Figure 1C). Although pesticides were intensively sprayed, many pepper plants showed viral disease symptoms (Figure 1D). To identify microorganisms in the overwintering pepper fruits, we extracted total DNA and total RNA from the same sample and used it for library preparation. DNA libraries were targeted to identify bacteria and DNA viruses, whereas RNA libraries were targeted for fungi and RNA viruses. From three different samples, we generated three DNA (D48, D63, and D89) and RNA (R48, R63, and R89) libraries. We obtained 12,265,600 reads (R63) to 39,586,002 reads (D48) (Table S1). The number of sequenced reads for the three DNA libraries was about three times higher than that for the three RNA libraries.

**Figure 2:**
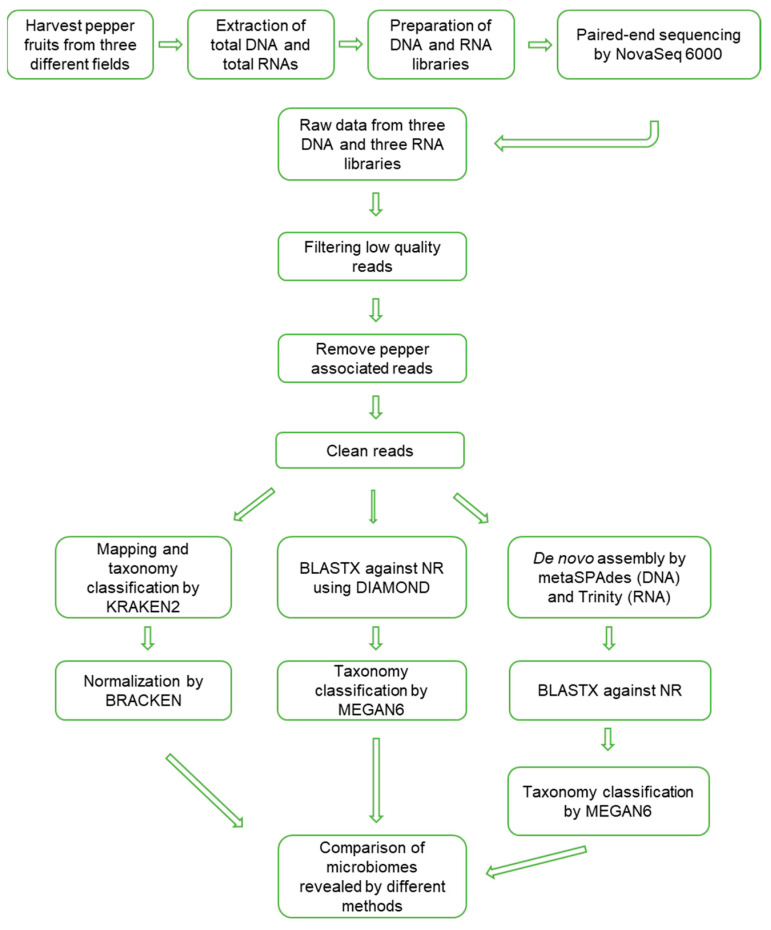
Schematic diagram to analyze microbiomes in overwintering pepper fruits using metagenomics and metatranscriptomics.

The data analyses to reveal the microbiomes were processed, as shown in Figure 2. Firstly, raw data were trimmed to filter high-quality reads. The portion of trimmed reads ranged from 8.47% (D89) to 21.07% (D48) (Table S1). Trimmed reads were subjected to mapping on the pepper nuclear, chloroplast, and mitochondrial genomes. The proportion of pepper-associated reads ranged from 42.31% (D48) to 81.27% (D89). After removing low-quality reads and pepper-associated reads, the proportion of filtered reads ranged from 17.54% to 53.05%.

### 2.2. Identification of Microorganisms by Three Different Methods

The filtered clean reads were subjected to microbial identification using the KRAKEN2 v.2.1.1 (KRAKEN2_R) and MEGAN6 v.6.20.19 (MEGAN6_R) programs. The proportion of classified reads by KRAKEN2_R ranged from 30.51% (R63) to 79.05% (D89), whereas the proportion of classified reads by MEGAN6_R ranged from 14.97% (D89) to 76.12% (D48) (Table S2). Although we removed pepper-associated reads, there were still plant-associated reads (Table S2). The number of plant-associated reads by KRAKEN2_R ranged from 936 (D63) to 5134 (R89), whereas the number of plant- and animal-associated reads ranged from 3655 (D89) to 97,458 (R48). KRAKEN2_R identified protozoa-associated reads ranging from 78 (D63) to 367 (R89), whereas MEGAN6_R identified several animal-associated reads, such as *Brugia timori* (612 reads in R48), *Onchocerca ochengi* (470 reads in R48), and *Lucilia cuprina* (354 reads in R63). In addition, MEGAN6 revealed many archaea-associated reads only from three RNA libraries (1577 to 4239 reads) (Table S2). The number of fungi-associated reads by MEGAN6_R (1,192,193 reads) was higher than that by KRAKEN2_R (912,681 reads). By contrast, the number of bacteria-associated reads by KRAKEN2_R (12,412,185 reads) was 1.6 times higher than that by MEGAN6_R (7,745,767 reads). The number of virus-associated reads by KRAKEN2_R (297,670) was slightly higher than that by MEGAN6_R (283,367 reads).

Moreover, we de novo assembled DNA and RNA raw data using the metaSPAdes and Trinity programs followed by BLASTX search and MEGAN6 analysis referred to as MEGAN6_A (Table S3). By MEGAN6_A, we identified 129,498 bacterial, 65,170 fungal, and 670 viral contigs (Table S2).

### 2.3. Rarefaction Analysis

**Figure 3:**
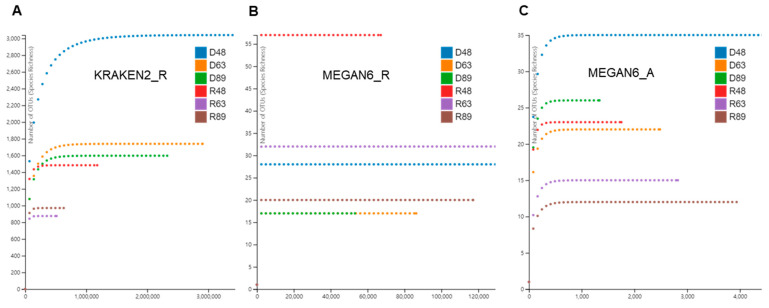
Rarefaction curves of microbiomes in overwintering pepper fruits using three different analytical methods: KRAKEN2_R (**A**), MEGAN6_R (**B**), and MEGAN6_A (**C**).

Based on the identified species for bacteria, fungi, and viruses, we examined rarefaction curves for individual libraries analyzed by KRAKEN2_R, MEGAN6_R, and MEGAN6_A (Figure 3). In general, the rarefaction curves of microorganism species identified by KRAKEN2 were much higher than those by MEGAN6 (Figure 3). The diversity of microbial species in D48 revealed by KRAKEN2_R was the highest among the six libraries followed by D63, D89, and R48 (Figure 3A). By MEGAN6_R, R48 was the highest among the six libraries followed by R63, D48, and R89 (Figure 3B). The diversity of microbial species by MEGAN6_A was the lowest among the three different methods (Figure 3C). Based on MEGAN6_A, D48 showed the highest diversity of microbial species followed by D89, R48, and D63.

### 2.4. Alpha Diversity of Identified Microorganisms

**Figure 4:**
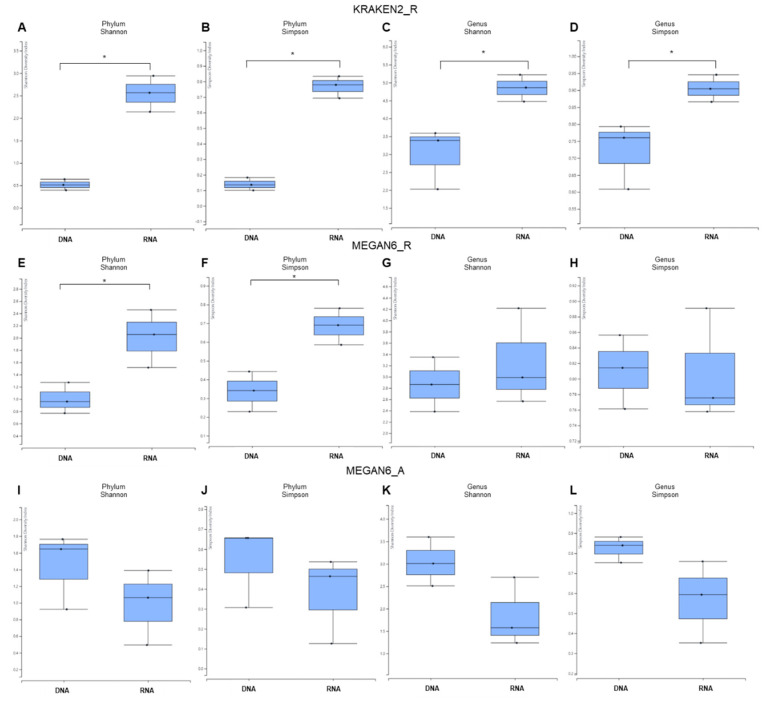
Alpha diversity of identified microbiomes at phylum and genus levels between DNA and RNA libraries. Alpha diversity of microorganisms identified by KRAKEN2_R (**A**–**D**), MEGAN6_R (**E**–**H**), and MEGAN6_A (**I**–**L**) using two different diversity indexes (i.e., Shannon and Simpson). The statistical test carried out was the Wilcoxon rank-sum test (nonparametric). * indicates a *p*-value less than 0.05.

Based on the phylum and genus, we examined the alpha diversity for identified microorganisms between DNA and RNA libraries (Figure 4).

In general, the alpha diversity in RNA libraries was much higher than that in DNA libraries by KRAKEN2_R (Figure 4A–D). By both the Shannon and Simpson diversity indexes, the difference in α diversity between the DNA and RNA libraries was significant (*p*-value = 0.04953) at the phylum and genus levels. Using MEGAN6_R, the α diversity for RNA libraries was again higher than that of DNA libraries at only the phylum level, and the α diversity between the DNA and RNA libraries was significantly different at only the phylum level (Figure 4E,F). We did not observe any significant difference in α diversity between the DNA and RNA libraries at the genus level (Figure 4G,H). By MEGAN6_A, the α diversity in the DNA libraries was higher than that in the RNA libraries at both the phylum and genus levels (Figure 4I–L). There was no statically significant difference in α diversity between the DNA and RNA libraries at either the phylum or genus level.

### 2.5. Comparison of Classified and Unclassified Reads in Six Libraries and Methods

**Figure 5:**
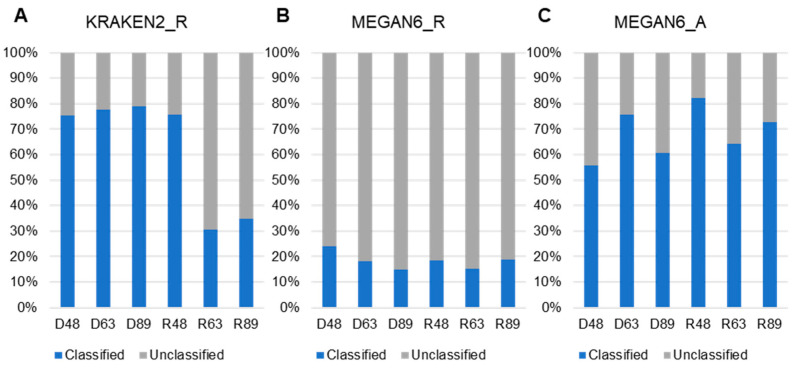
Proportion of classified and unclassified reads or contigs in each library using KRAKEN2_R (**A**), MEGAN6_R (**B**), and MEGAN6_A (**C**).

We examined the proportion of classified and unclassified reads (unmapped reads to any known organisms) for KRAKEN2_R and MEGAN6_R or contigs for MEGAN6_A (Figure 5). By KRAKEN2_R, the three DNA libraries and R48 samples showed a high proportion of classified reads ranging from 75.4% (D48) to 79.05% (D89), whereas the proportion of classified reads in two RNA libraries was very low (i.e., 30.5% (R63) and 34.8% (R89)) (Figure 5A). By MEGAN6_R, the proportion of classified reads was very low, ranging from 18% (D63) to 23.87% (D48) regardless of library type (Figure 5B). The proportion of classified contigs ranging from 56.4% (D48) to 84.46% (R48) was much higher than that of unclassified contigs by MEGAN6_A (Figure 5C).

### 2.6. Comparison of Proportion for Three Different Microorganisms

**Figure 6:**
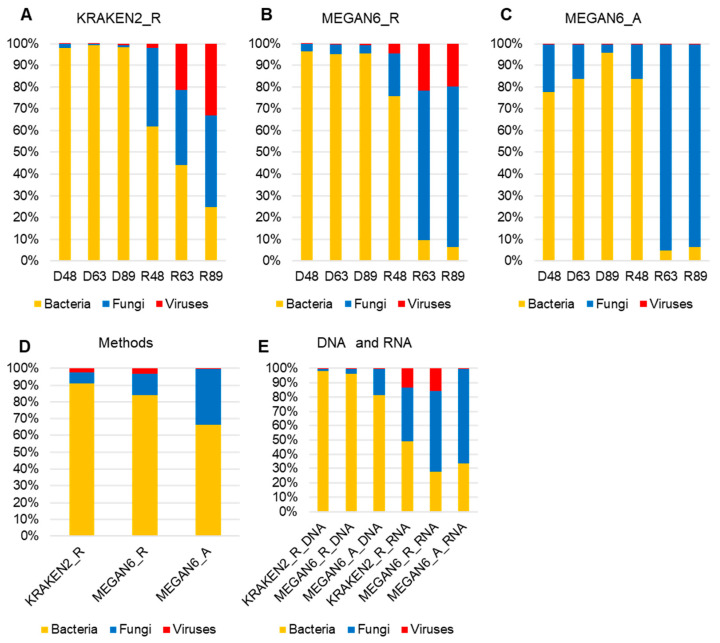
Proportion of three different microbial groups (i.e., bacteria, fungi, and viruses) in each library identified using KRAKEN2_R (**A**), MEGAN6_R (**B**), and MEGAN6_A (**C**). Comparison of identified microbial groups by different methods (**D**) and library types (i.e., DNA and RNA) (**E**).

We examined the proportion of microorganisms such as bacteria, fungi, and viruses in each library identified by the three methods (Figure 6A,C). By all three methods, bacteria were dominantly identified in all three DNA libraries, whereas fungi were dominantly identified in the RNA libraries. The proportion of viruses was very high by KRAKEN2_R and MEGAN6_R. In particular, two RNA libraries, R63 and R89, possessed a high number of virus-associated reads (Figure 6A,B). However, the proportion of virus-associated contigs was very low by MEGAN6_A (Figure 6C). Among the three different methods, the proportion of bacteria was the highest by KRAKEN2_R, whereas the proportion of fungi was the highest by MEGAN6_A (Figure 6D). When we compared the DNA and RNA libraries, the proportion of bacteria was very high in the DNA libraries as compared to the RNA libraries regardless of method (Figure 6E). Moreover, the proportion of fungi and viruses was very high in the RNA libraries regardless of method (Figure 6E).

### 2.7. Comparison of Number of Identified Microorganisms by Three Different Methods

**Figure 7:**
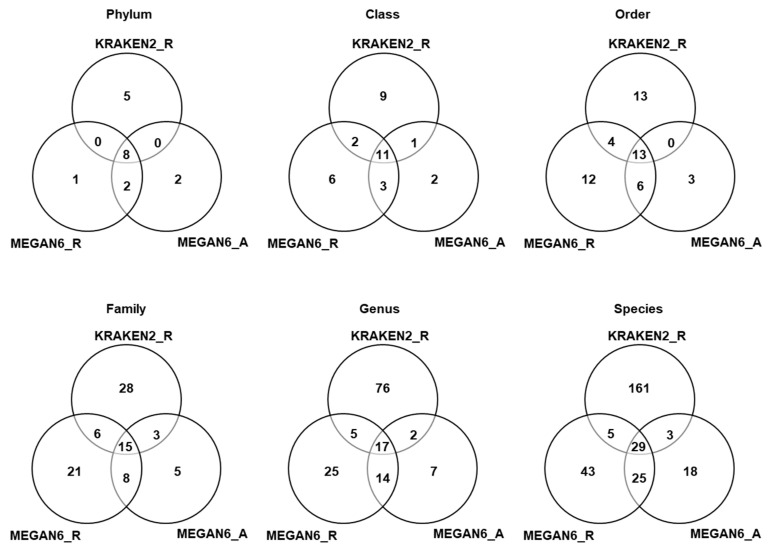
Comparison of identified microorganisms at different taxonomy levels (i.e., phylum, class, order, family, genus, and species) by three different analytical methods (i.e., KRAKEN2_R, MEGAN6_R, and MEGAN6_A).

We compared the number of identified microorganisms such as bacteria, fungi, and viruses among the three different methods based on six different taxonomies (Tables S4–S6 and Figure 7). In the case of KRAKEN2_R identifying more than 3000 bacterial species, we only selected the top 100 bacterial species to simplify the comparison (Table S4). According to phylum, eight phyla (three, four, and four phyla for bacteria, fungi, and viruses) were commonly identified among the three different methods (Figure 7). Moreover, 11 classes, 13 orders, 15 families, 17 genera, and 29 species were commonly identified among the three different methods. Based on phylum, the number of phyla identified by KRAKEN2 _R (13 phyla) was higher than that identified by MEGAN6 using raw data (11 phyla) and MEGAN6 using assembled contigs (11 phyla). However, the number of species identified by KRAKEN2_R (198 species) was much higher than that identified by MEGAN6_R (103 species) and MEGAN6_A (76 species).

### 2.8. Comparison of Identified Bacterial Phyla and Genera

**Figure 8:**
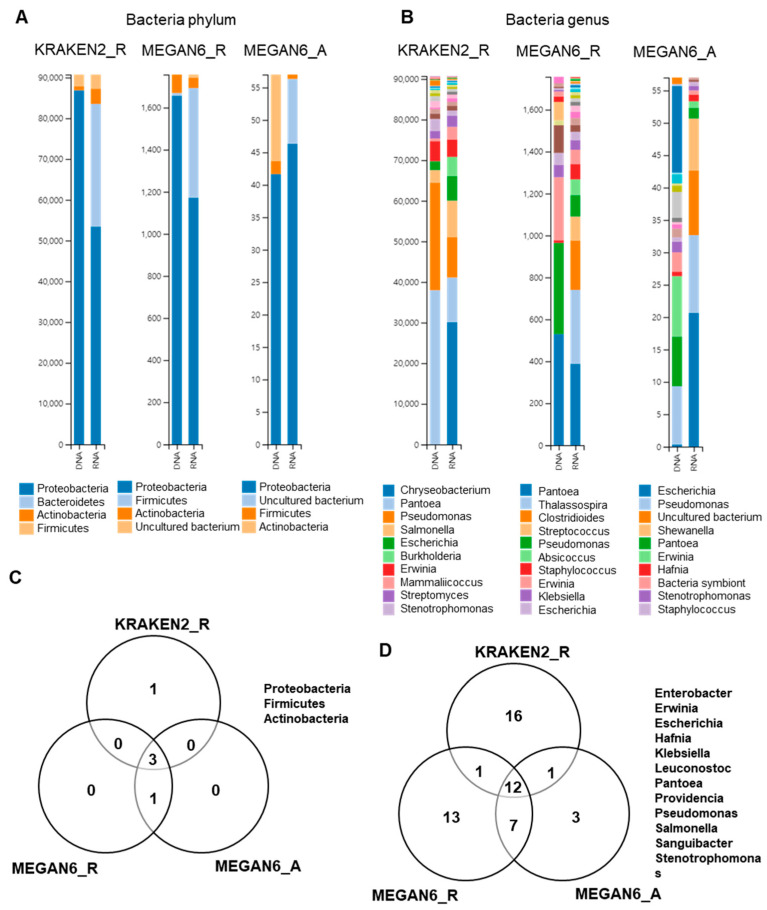
Comparison of identified bacteria at phylum and genus levels by three different analytical methods (i.e., KRAKEN2_R, MEGAN6_R, and MEGAN6_A). Proportion of identified bacterial phyla (**A**) and genera (**B**). Commonly identified phyla (**C**) and genera (**D**) among three different analytical methods.

We examined bacterial phyla and genera identified by three different methods (Figure 8). Four different phyla were identified by the three methods (Figure 8A), and three bacterial phyla (i.e., *Proteobacteria*, *Actinobacteria*, and *Firmicutes*) were commonly identified (Figure 8B). Of them, *Proteobacteria* was dominantly identified in both the DNA and RNA libraries. By KRAKEN2_R, *Pantoea* and *Pseudomonas* were dominantly identified in the DNA libraries, whereas *Chryseobacterium*, *Pantoea*, and *Pseudomonas* were dominantly identified in the RNA libraries (Figure 8B). By MEGAN6_R, *Pantoea*, *Pseudomonas*, and *Erwinia* were dominantly identified in the DNA libraries, whereas *Pantoea*, *Thalassospira*, and *Clostridioides* were dominantly identified in the RNA libraries. Using MEGAN6_A, *Escherichia*, *Pantoea*, and *Erwinia* were identified from the DNA libraries, while *Escherichia*, *Pseudomonas*, and an uncultured bacterium were identified from the RNA libraries. At least 12 bacterial genera were commonly identified from the three different methods (Figure 8D); however, many bacterial genera were only identified by a specific analytical method.

### 2.9. Comparison of Identified Fungal Phyla and Genera

**Figure 9:**
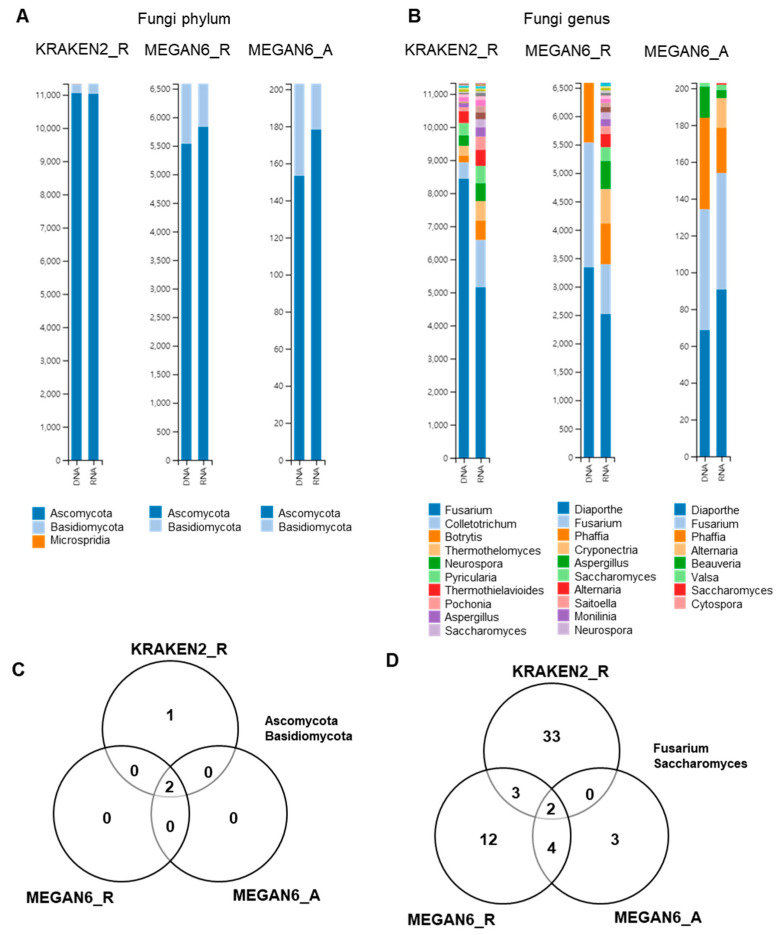
Comparison of identified fungi at phylum and genus levels by three different analytical methods (i.e., KRAKEN2_R, MEGAN6_R, and MEGAN6_A). Proportion of identified fungal phyla (**A**) and genera (**B**). Commonly identified phyla (**C**) and genera (**D**) among three different analytical methods.

Next, we compared the identified fungal phyla and genera by the three different methods (Figure 9). *Ascomycota* was the dominant fungal phylum followed by *Basidiomycota* by all three different methods (Figure 9A). At the genus level, the diversity of identified fungal genera was higher in the RNA libraries as compared to in the DNA libraries (Figure 9B). *Microsporidia* was only identified by KRAKEN2_R (Figure 9C). The most dominant fungal genus was *Fusarium* followed by *Colletotrichum* by KRAKEN2_R regardless of library type. Interestingly, the major fungal genera by both MEGAN6_R and MEGAN6_A were *Diaporthe* and *Fusarium* (Figure 8C). *Diaporthe* was not identified by KRAKEN2_R. Among the three different methods, two fungal genera, *Fusarium* and *Saccharomyces*, were commonly identified (Figure 8D).

### 2.10. Comparison of Identified Viral Phyla and Genera

**Figure 10:**
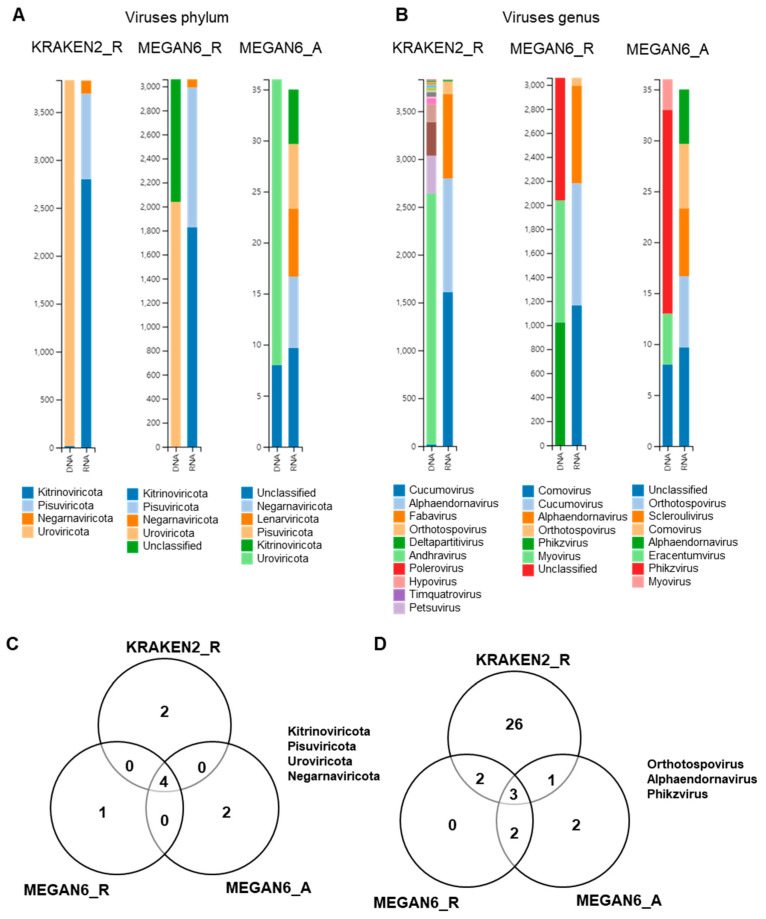
Comparison of identified viruses at phylum and genus levels by three different analytical methods (i.e., KRAKEN2_R, MEGAN6_R, and MEGAN6_A). Proportion of identified viral phyla (**A**) and genera (**B**). Commonly identified phyla (**C**) and genera (**D**) among three different analytical methods.

By KRAKEN2_R, we identified four viral phyla, which were *Uroviricota* only from the DNA libraries and *Kitrinoviricota*, *Pisuviricota*, and *Negarnaviricota* from the RNA libraries (Figure 10A). By MEGAN6_R, five viral phyla were identified. Of them, *Uroviricota* and an unclassified viral phylum were identified from the DNA libraries, and three other viral phyla were identified from the RNA libraries. By MEGAN6_A, *Uroviricota* and an unclassified viral phylum were identified from the DNA libraries, and five different viral phyla were identified from the RNA libraries. Four different viral phyla, *Kitrinoviricota*, *Pisuviricota*, *Uroviricota*, and *Negarnaviricota*, were commonly identified by the three different methods (Figure 9C). At the genus level, many different viral genera were identified by KRAKEN2_R (Figure 9B). The most abundant viral genus in the DNA libraries was andhravirus, whereas cucumovirus, alphaendornavirus, and fabavirus were frequently identified in the RNA libraries by KRAKEN2_R. By MEGAN6_R, three genera, myovirus, phikzvirus, and an unclassified viral genus, were identified from the DNA libraries, while comovirus, cucumovirus, and alphaendornavirus were identified from the RNA libraries. By MEGAN6_A, the most abundant viral genus in the DNA libraries was phikzvirus, while five different viral genera were identified from the RNA libraries. Three different viral genera, orthotospovirus, alphaendornavirus, and phikzvirus, were commonly identified by the three different methods (Figure 9D). Of the three different methods, KRAKEN2_R identified the highest number of viral genera.

We de novo assembled several viruses infecting pepper from RNA-seq data (Table S7). They were hot pepper alphaendornavirus (HPEV) isolate 48 (MW815616), CMV isolate R48 with RNA1 fragment (MW773728), BBWV2 isolate 48 with RNA2 fragment (MW815615), CMV isolate R63 with three RNA fragments (MW815617–MW815619), BBWV2 isolate R63 with two RNA fragments (MW815620 and MW815621), CMV isolate R89 with three RNA fragments (MW815622–MW815624), and BBWV2 isolate R89 (MW815625–MW815626). In addition, we identified partial sequences associated with pepper cryptic virus 1 (PCV1) (125 reads), pepper cryptic virus 2 (PCV2) (141 reads), and TSWV (8419 reads) by KRAKEN2_R. The reads assigned to zucchini lethal chlorosis orthotospovirus (24 reads) and chrysanthemum stem necrosis orthotospovirus (111 reads) were reads derived from TSWV (8419 reads). Similarly, reads associated with tomato aspermy virus (884 reads) and gayfeather mild mottle virus (14 reads) were derived from CMV. In addition, we obtained partial sequences (20 contigs) associated with sclerotinia scleroulivirus 1 by MEGAN6_A and valsa ceratosperma hypovirus 1 (29 reads) by KRAKEN2_R.

**Figure 11:**
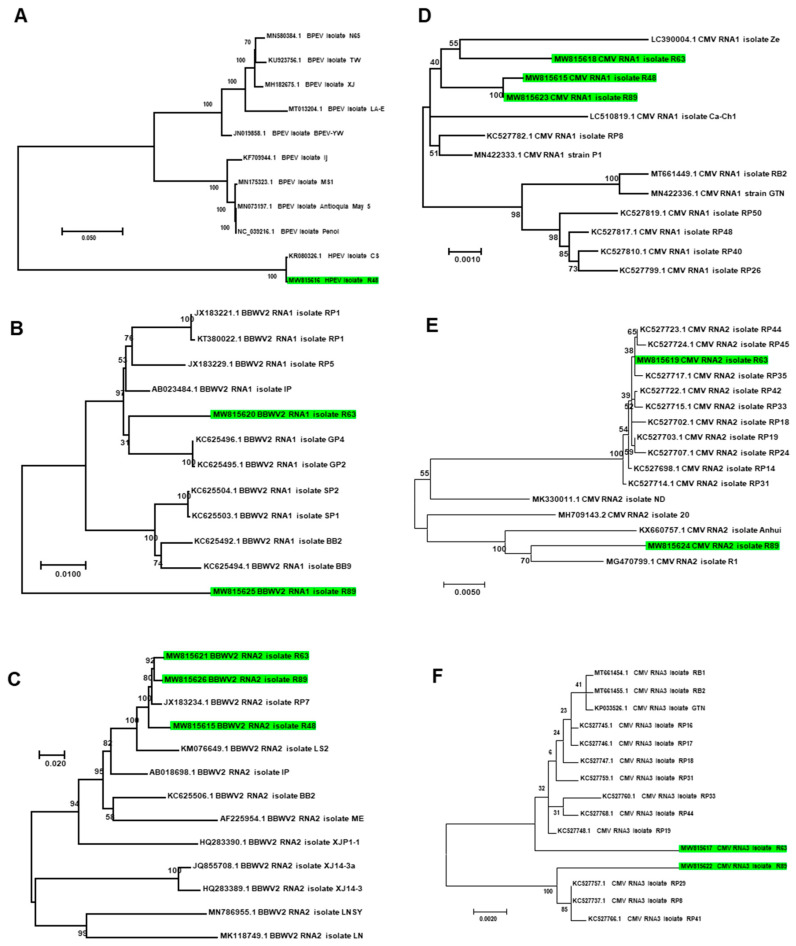
Phylogenetic relationships of assembled viral genomes. Phylogenetic trees for HPEV (**A**), BBWV2 RNA1 (**B**), BBWV2 RNA2 (**C**), CMV RNA1 (**D**), CMV RNA2 (**E**), and CMV RNA3 (**F**) were constructed using the maximum likelihood method using the MEGA7 program. Green colored boxes indicate viral genome fragments assembled from this study.

The phylogenetic tree showed that HPEV isolate R48 was closely related with HPEV isolate CS in Korea (Figure 11A). Of the two assembled RNA1 fragments for BBWV2, BBWV2 RNA1 isolate R63 was distantly related to known BBWV2 RNA1 isolates from Korea, while BBWV2 RNA1 isolate R63 was grouped together with other isolates from Korea (Figure 11B). In the case of RNA2 of BBWV2, all three isolates in this study were grouped together with other isolates from Korea (Figure 11C). CMV was composed of three RNA fragments. The three RNA1 fragments in this study were closely related with other isolates from Korea (Figure 11D); however, RNA2 and RNA3 for isolate R63 were genetically different from those for isolate R89 (Figure 11E,F).

Moreover, we identified bacteriophages such as unclassified eracentumvirus (15 contigs), pseudomonas phage (60 contigs), Erwinia phage (nine contigs), and unknown bacteriophage species (29 contigs) by MEGAN6_A. BLASTX search using assembled contig-associated bacteriophages revealed that most viral sequences derived from bacteriophages were novel. Several bacteriophages were identified by KRAKEN2_R. Of them, the most abundant bacteriophage was staphylococcus virus Andhra (19,009 reads) followed by Edwardsiella virus pEtSU (1411 reads) and pseudomonas phage OBP (1227 reads).

### 2.11. Classification of Identified Microbial Species by KRAKEN2_R

**Figure 12:**
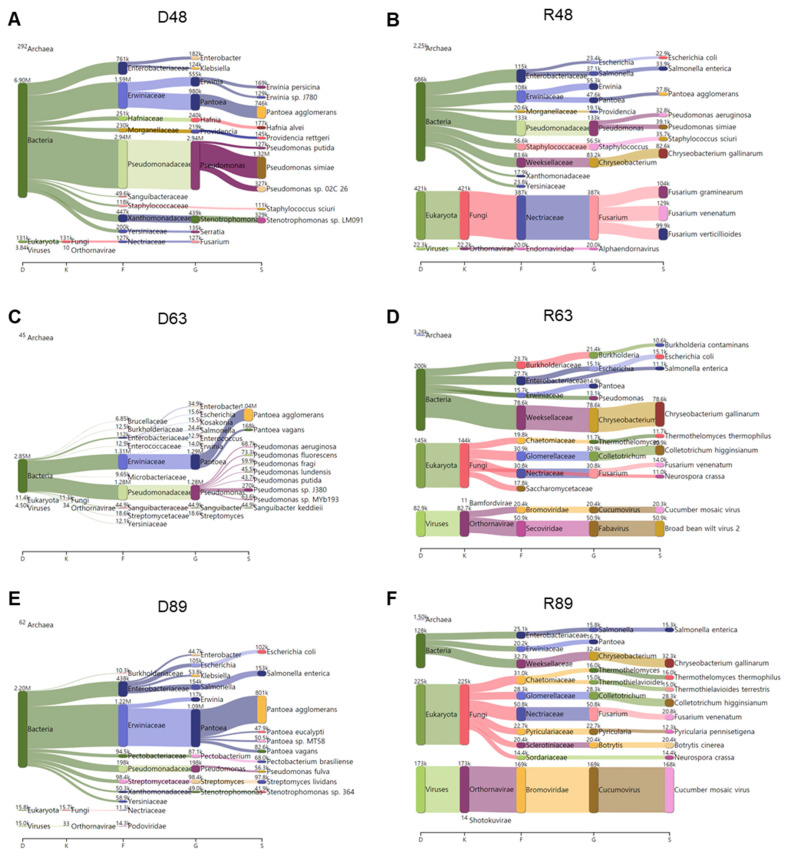
Major microbiomes identified from six different libraries using KRAKEN2_R. Sankey diagram displays major microbiomes in D48 (**A**), R48 (**B**), D63 (**C**), R63 (**D**), D89 (**E**), and R89 (**F**) libraries with abundance of microbial species.

KRAKEN2_R identified the highest number of microbial species among the three methods. Therefore, we examined the major microbial species in each library identified by KRAKEN2_R using a Sankey diagram displaying five taxonomies: domain, kingdom, family, genus, and species (Figure 12). In D48, bacteria were abundantly identified. Of them, *Pseudomonas simiae*, *Pantoea agglomerans*, and *Stenotrophomonas* sp. LM091 were major bacterial species (Figure 12A). In R48, not only bacteria but also fungi were dominantly identified. The most abundant bacterial species in R48 was *Chryseobacterium gallinarum*, and three different *Fusarium* species (i.e., *F. graminearum*, *F. venenatum*, and *F. verticillioides*) were identified (Figure 12B). In D63, we identified mostly bacteria that were assigned to two major families: *Erwiniaceae* and *Pseudomonadaceae* (Figure 12C). *Pantoea agglomerans* was the most abundant microbial species in D63. In addition, several *Pseudomonas* species were identified in D63. In R63, we identified several microbial species for bacteria, fungi, and viruses (Figure 12D). For example, in R63, *Chryseobacterium gallinarum* was the dominant bacterial species, while *Colletotrichum higginsianum* was the dominant fungal species. In addition, we identified a high number of viral reads associated with CMV and BBWV2 in R63. In D89, *Pantoea agglomerans* was the most dominant microbial species followed by *Salmonella enterica* and *Escherichia coli* (Figure 12E). In R89, the most abundant microbial species was CMV (Figure 12F). In addition, *Chryseobacterium gallinarum* was the dominant bacterial species, while many different fungal species such as *Colletotrichum higginsianum*, *Fusarium venenatum*, and *Botrytis cinerea* were identified in R89.

## 3. Discussion

In this study, we used two different types of libraries for microbiome study. The DNA shotgun library was derived from random DNA strands of the sample obtained by total DNA extraction without any deletion of the contaminants. Thus, the DNA shotgun library might have contained a high portion of host DNA as well as other microbial DNA sequences. As we expected, the portion of pepper genome (nucleus, chloroplast, and mitochondrion)-associated reads was very high (e.g., 42.31–81.27%). Interestingly, the D48 library with the lowest portion of pepper genome-associated reads contained a high level of bacterial reads. Regardless of microbiome analytical method, the DNA shotgun libraries very efficiently revealed bacterial populations in the sample. However, we found that the portions of fungi- and virus-associated reads in the three DNA libraries were very low. This result suggests that DNA shotgun sequencing might not be appropriate for mycobiome and virome study except for viruses with DNA genomes such as bacteriophages. DNA shotgun sequencing successfully provided a comprehensive overview of bacterial diversity and abundance in the overwintering pepper fruits.

To date, several types of RNA libraries have been used for microbiome study. The selection of RNA library type depends on the purpose of the experiments. Two well-known RNA library types are total RNA and mRNA libraries. Total RNA is useful to study both coding and noncoding RNAs of all organisms in each sample, and total RNA extraction followed by rRNA depletion is usually used. Total RNA libraries without rRNA have been widely used for microbiome studies such as those on bacteria, fungi, and viruses. By contrast, mRNA libraries using poly(A) enrichment represent only a small portion of the total RNAs but are the most efficient tool for a wide range of transcriptome analyses including gene expression analysis. In this study, we used the mRNA libraries instead of the total RNA libraries deleted rRNA. We selected the mRNA libraries for the detection of microorganisms in this study for several reasons. Of the three different types of microorganisms (bacteria, fungi, and viruses), the major target of this study was fungi infecting the overwintering pepper fruits. Therefore, we used the mRNA libraries to facilitate the detection of fungal sequences with poly(A) tails. Of course, we obtained a portion of pepper mRNAs ranging from 63.62% to 68.55%. However, the proportion of fungal reads in the RNA libraries was high enough to reveal fungal populations in the sample. As a result, the mRNA libraries were much better to identify diverse fungal species with a high number of reads as compared to DNA libraries possessing a small number of fungal reads.

Although viruses were not our major targets in this study, we identified many kinds of viruses infecting bacteria and pepper. The most abundant viruses infecting pepper were CMV (7 RNA fragments), BBWV2 (5 RNA fragments), and HPEV (1 RNA fragment), in which several nearly complete genome sequences were *de novo* assembled from RNA-seq. Moreover, we identified PCV1, PCV2, and TSWV based on the reads and assembled contigs. We found that some viral species were wrongly assigned by KRAKEN2_R. For example, reads assigned to zucchini lethal chlorosis orthotospovirus (24 reads) and chrysanthemum stem necrosis orthotospovirus (111 reads) should have been taxonomically assigned to TSWV. Similarly, tomato aspermy virus (884 reads) and gayfeather mild mottle virus (14 reads) should have been assigned to CMV. Based on these results, we concluded that KRAKEN2_R identified the highest number of viral species among the three methods, but some viral species were wrongly assigned. By contrast, MEGAN6_R and MEGAN6_A could not identify some viruses infecting pepper such as PCV1 and PCV2. Therefore, it is noteworthy that each analytical method has its own advantages and disadvantages. Although we compared the three analytical methods based on the plant viruses infecting pepper that have been well characterized, the taxonomy results using those methods might be very similar for the identification of bacteria and fungi. It seems that the identification of microorganisms by KRAKEN2_R might be reliable at the genus level rather than the species level.

Rarefaction results showed that KRAKEN2_R identified a high level of microbial diversity. Moreover, the proportion of classified reads by KRAKEN2_R was the highest among the three methods. By contrast, the proportion of classified reads was very low in MEGAN6_R, suggesting that the non-redundant protein database for BLASTX search was not sufficient for the identification of microorganisms. In addition, we found that KRAKEN2_R was the most efficient tool to identify bacteria. These results suggest that KRAKEN2_R can be usefully applied for the study of plant bacteria.

It was noteworthy that only a small portion of microbial species was commonly identified by the three different analytical methods. This result suggests that different analytical methods and databases resulted in different sets of microbial identification. For instance, KRAKEN2_R did not identify *Diaporthe*, although *Diaporthe* was the dominant fungal genus in both DNA and RNA samples by MEGAN6_R and MEGAN6_A. Moreover, we found that the commonly identified microorganisms by the three different methods were major microorganisms that were abundantly present in the sample.

The phyllosphere, including the leaves, stems, flowers, and fruits, is an important microbial habitat that plays critical roles in plant health [32]. However, fewer studies associated with microbiomes in the phyllosphere have been conducted as compared to those associated with microbiomes in the rhizosphere. In this study, using metagenomics and metatranscriptomics, we revealed that *Pseudomonas*, *Pantoea*, and *Erwinia* were frequently identified bacterial genera in the overwintering pepper fruits. Of them, two genera, *Pantoea* and *Erwinia*, have been identified as major bacterial genera of the fresh leaves of lettuce [33]. Similarly, recently, fresh fruits such as those of sweet pepper have been examined by bacterial 16S rRNA gene profiling [34]. The previous study revealed that the most abundant bacterial genera on the fresh sweet pepper in South Africa were associated with fungal antagonists such as *Acinetobacter*, *Agrobacterium*, and *Burkholderia*. *Pseudomonas simiae* was identified as the most abundant bacterial species in the D48 library. Similarly, a recent study showed that *Pseudomonas simiae* plays an important role in the biological control of *Verticillium dahliae* [35]. In addition, several studies have demonstrated the functional roles of *Pseudomonas simiae* in abiotic stress adaptation [36,37]. By contrast, *Pantoea agglomerans* was the most abundant bacterial species in the D63 and D89 libraries. *Pantoea agglomerans* has been known as a widespread epiphyte and commensal pathogenic plant bacterium causing gall formation [38]. In addition, *Pantoea agglomerans* is a frequently identified bacterial species in humans causing several human diseases [39]. Thus, *Pantoea agglomerans-*infected vegetables such as pepper fruits might be a threat to plant health. Interestingly, some bacterial species including *Chryseobacterium gallinarum* were identified only from RNA samples. A previous study reported that bacteria belonging to the *Chryseobacterium* genus were frequently identified in leaf salad vegetables [22].

Fungal pathogens are regarded as major problems for the production of high-quality pepper fruits. Three *Fusarium* species causing fruit rot diseases, *F. graminearum*, *F. venenatum*, and *F. verticillioides,* have been identified by RNA sequencing. Interestingly, we identified a high proportion of *Fusarium* from pepper fruits grown in the greenhouse (R48 library). Similarly, fruit rot disease by *Fusarium* species was reported in the pepper plants grown in a commercial greenhouse [40]. Moreover, we identified other fungal species causing pepper fungal diseases such as *Colletotrichum higginsianum* (pepper anthracnose), *Botrytis cinerea* (gray mold disease), and *Diaporthe* (fruit decay) [5,9,41]. In particular, this is the first report of *Diaporthe* species causing fruit decay in pepper fruits in the Republic of Korea.

In this study, we identified several bacteriophages by DNA sequencing; however, the most abundant viruses were RNA viruses infecting pepper identified by RNA sequencing. Many previous studies have shown that geminiviruses are the major viruses in many Asian countries such as India and Vietnam [42,43]. However, we did not identify any geminiviruses infecting pepper plants. Although different viruses were co-infected in the pepper fruits, the dominant virus differed in each sample (e.g., HPEV in R48 and CMV in R89). Moreover, we could assemble viral genomes for three viruses and quantify the abundance of individual viruses by RNA sequencing. However, the possible relationship between viral disease symptoms and co-infected viruses should be further elucidated. In the case of RNA viruses composed of more than two RNA segments, the phylogenetic relationships between RNA segments differed. This result suggests that individual viral RNA fragments for the same virus might evolve differently.

The compositions of microorganisms in the three different regions were apparently different from each other. It seems that several factors such as the different pepper cultivar, different soil type, and different cultivation conditions might determine the composition of microorganisms. For example, HPEV is specifically identified in the 48 region and is known to be vertically transmitted by pollen [44]. Therefore, the pepper cultivar in the 48 region was different from that in the other two regions. By contrast, BBWV2 and CMV, which are transmitted by insects such as aphids, were identified in all three regions. Coinfection of CMV and BBWV2 in red pepper in Korea is very common and results in severe mosaic symptoms [45].

DNA sequencing did not distinguish between viable and dead microorganisms; however, the microorganisms identified by RNA sequencing could be viable [29]. For example, in the 48 region, the three bacterial genera *Erwinia*, *Pantoea*, and *Pseudomonas* were dominantly present. However, the RNA sequencing result showed that the proportions of those three bacterial genera were significantly reduced. Similarly, the proportion of *Pantoea agglomerans* was significantly reduced in R89 compared to D89. These results suggesting that many of the bacteria identified by DNA sequencing might be not viable.

Of the identified bacteria species, some might be contaminations. For instance, the two bacterial pathogens *Escherichia coli* and *Salmonella enterica* are well-known human pathogens rather than plant pathogens [46]. Identification of human pathogens in the pepper plants suggests the contamination of our collected samples from soils or other sources.

As shown in this study, overwintering pepper plants are reservoirs of diverse pathogenic microorganisms. Therefore, it is necessary to remove pepper plants before the winter season. Moreover, it is also desirable not to plant the same plant species in series since the soil in which pepper plants were cultivated will contain numerous pathogenic microorganisms.

## 4. Conclusions

In this study, we compared two different library types (DNA and mRNA libraries) and three different analytical methods for the identification of microbiomes in overwintering pepper fruits. Our results demonstrated that DNA sequencing might be useful for the identification of bacteria and DNA viruses such as bacteriophages, while mRNA sequencing might be beneficial for the identification of fungi and RNA viruses. Among the three analytical methods, KRAKEN2_R might be superior for the identification of microbial species—especially for bacteria—to other analytical methods. However, some microbial species with a low number of reads were wrongly assigned at the species level by KRAKEN2_R. For example, KRAKEN2_R identified the highest number of viral species among the three methods but some viral species were wrongly assigned. By contrast, MEGAN6_R and MEGAN6_A could not identify viruses with a small number of reads. The identification of microorganisms by KRAKEN2_R might be reliable at the genus level rather than at the species level. We revealed that *Pseudomonas*, *Pantoea*, and *Erwinia* were frequently identified bacterial genera in overwintering pepper fruits. Three Fusarium species causing fruit rot diseases—*F. graminearum*, *F. venenatum*, and *F. verticillioides*—have been identified by RNA sequencing. Moreover, we identified other fungal species that cause pepper fungal diseases such as *Colletotrichum higginsianum*, *Botrytis cinerea*, and *Diaporthe*. By mRNA sequencing, we identified most well-known RNA viruses infecting pepper in Korea including CMV, BBWV2, HPEV, PCV1, PCV2, and TSWV. Furthermore, we found that different analytical methods and databases resulted in different sets of microbial identification. The databases for bacteria and viruses were better established compared to the fungal database with limited genome data. We carefully suggest that different library types and analytical methods with proper databases should be applied for the purpose of microbiome study.

## 5. Materials and Methods

### 5.1. Plant Materials and Nucleic acid Extraction

We collected pepper fruits from three different regions referred to as Regions 48, 63, and 89 according to the administration area in Hoengseong, Korea. We did not use three biological repetitions. The pepper plants from Region 48 were grown in a greenhouse, whereas the pepper plants from Regions 63 and 89 were grown in open fields. All pepper plants were all planted in May. Three different pepper fruits from the same plant were randomly collected from each region and immediately frozen in liquid nitrogen. The three whole pepper fruits from each region were ground in the presence of liquid nitrogen using a pestle and mortar. The same ground pepper powder from each region was used for total DNA extraction and total RNA extraction. Total DNA was extracted using the DNeasy Plant Mini Kit (Qiagen, Hilden, Germany), whereas total RNA was extracted using the RNeasy Plant Mini Kit (Qiagen, Hilden, Germany) according to the manufacturer’s instructions.

### 5.2. Library Preparation and NGS

After performing quality control, we prepared three DNA libraries to identify bacteria and DNA viruses and three RNA libraries to identify fungi and RNA viruses. The DNA libraries were prepared using TruSeq DNA Sample Prep Kits, and the RNA libraries were prepared using the TruSeq Stranded mRNA Library Prep Kit according to the manufacturer’s instructions (Illumina, San Diego, CA, USA). All libraries were ligated to multiple indexing adapters and paired-end (100 bp × 2) sequenced by the NovaSeq 6000 System from Macrogen (Seoul, Korea). All raw data were deposited in the National Center for Biotechnology Information (NCBI)’s SRA database with respective accession numbers (Table S1).

### 5.3. Quality Trimming and Removal of Pepper-Associated Reads

The obtained raw data were subjected to quality control to remove low-quality bases (Phred quality score < 20) and short reads less than 50 bp using the BBDuk v.37.33 program (https://jgi.doe.gov/data-and-tools/bbtools/bb-tools-user-guide/bbduk-guide/) (accessed on 11 November 2020). We removed pepper-associated reads such as the nuclear genome (ftp://ftp.solgenomics.net/genomes/Capsicum_annuum/C.annuum_UCD10X/Capsicum_annuum_UCD10X_v1.0.fasta.gz) (accessed on 11 November 2020), chloroplast genome (NC_018552.1), and mitochondrial genome (NC_024624.1) using BBDuk v.37.33. The filtered reads were finally used for microbiome analyses.

### 5.4. Microbiome Analysis by KRAKEN2 and BRACKEN Using Raw Data

The filtered paired-end sequence reads in FASTQ format were subjected to KRAKEN2 analysis against the PlusPFP database containing archaea, bacteria, viral, plasmid, human, UniVec_core, protozoa, fungi, and plant (https://benlangmead.github.io/aws-indexes/k2) (accessed on 11 November 2020). The results from the KRAKEN2 analysis were subject to BRACKEN to estimate the abundance of identified species [47]. The KRAKEN2/BRACKEN results were analyzed by the Pavian program (https://fbreitwieser.shinyapps.io/pavian/) (accessed on 11 November 2020) to classify the metagenomic and metatranscriptomic results. The results from KRAKEN2/BRACKEN using raw data were named KRAKEN2_R.

### 5.5. Microbiome Analysis by BLASTX and MEGAN6 Using Raw Data

For each library, two paired-end FASTQ files were merged and converted to FASTA files using BBDuk v.37.33. The merged FASTA files were subjected to BLASTX (E-value less than 0.001) against non-redundant protein database (NR) v.20210102 using DIAMOND, producing DAA files [48]. The DAA files were imported to MEGAN6 for taxonomy assignment. The results from BLASTX and MEGAN6 using raw data were named MEGAN6_R.

### 5.6. Microbiome Analysis by BLASTX and MEGAN6 Using Assembled Contigs

The three sets of filtered DNA raw data were *de novo* assembled by metaSPAdes ver. 3.15.2 [49], whereas the three sets of filtered RNA raw data were *de novo* assembled by Trinity v.2.7.8a [50]. The assembled contigs were subject to BLASTX (E-value less than 0.001) against non-redundant protein database (NR) v.20210102 using DIAMOND, producing DAA files. The DAA files were imported to MEGAN6 for taxonomy assignment. The results from BLASTX and MEGAN6 using assembled contigs were named MEGAN6_A.

### 5.7. Microbial Taxonomy Assignment

We only extracted taxonomy results for microorganisms such as bacteria, fungi, and viruses from the three different methods of KRAKEN2_R, MEGAN6_R, and MEGAN6_A. The number of assigned reads for KRAKEN2_R and MEGAN6_R or contigs for MEGAN6_A was manually modified according to the seven main taxonomic ranks: kingdom, phylum, class, order, family, genus, and species. Rarefaction curves were calculated by the vegan package using only microorganisms based on the identified species [51]. Alpha diversity using two different indexes, Shannon and Simpson, was calculated based on the phylum and species for all identified microorganisms using the vegan package. A nonparametric test (Wilcoxon rank-sum) was conducted to test whether there was a difference in microorganisms between the DNA and RNA libraries using tools on the Mian website (https://miandata.org/) (accessed on 11 November 2020).

## Data Availability

All raw data in this study are publicly available in NCBI’s SRA database with the following accession numbers: SRR13319506–SRR13319511.

## Supplementary Materials

The following are available online at https://www.mdpi.com/article/10.3390/ijms22126202/s1.
